# Comparison of ankle-brachial index (ABI) measured by an automated oscillometric apparatus with that by standard hand-held doppler in patients with Type-2 diabetes

**DOI:** 10.12669/pjms.35.4.30

**Published:** 2019

**Authors:** Shair Zaman Khan, Awn-Bin-Zafar, Nazish Waris, Zahid Miyan, Muhammad Saif Ulhaque, Asher Fawwad

**Affiliations:** 1 Dr. Shair Zaman Khan, FCPS, Endocrine Fellow (BIDE). Baqai Institute of Diabetology and Endocrinology (BIDE), Baqai Medical University (BMU), Karachi, Pakistan; 2 Dr. Awn Bin Zafar, MCPS, M.D. Assistant Professor, Department of Medicine (BMU), Consultant Physician (BIDE) Baqai Institute of Diabetology and Endocrinology (BIDE), Baqai Medical University (BMU), Karachi, Pakistan; 3 Mrs. Nazish Waris, M.Phil., Ph.D. scholar, Research Officer, Research Department (BIDE) Baqai Institute of Diabetology and Endocrinology (BIDE), Baqai Medical University (BMU), Karachi, Pakistan; 4 Dr. Zahid Miyan, M.D. Assistant Professor, Department of Medicine (BMU), Consultant Physician (BIDE) Baqai Institute of Diabetology and Endocrinology (BIDE), Baqai Medical University (BMU), Karachi, Pakistan; 5 Dr. Muhammad Saif Ulhaque, MS (Dia., Endo), Registrar, Department of Medicine (BIDE) Baqai Institute of Diabetology and Endocrinology (BIDE), Baqai Medical University (BMU), Karachi, Pakistan; 6 Prof. Asher Fawwad, Ph.D. Chairman and Head of Department of Biochemistry (BMU), Research Director (BIDE). Baqai Institute of Diabetology and Endocrinology (BIDE), Baqai Medical University (BMU), Karachi, Pakistan

**Keywords:** Ankle-brachial index, Type-2 diabetes, Peripheral arterial disease

## Abstract

**Objective:**

To compare the difference between an automated oscillometric ABI measurement as compared to standard hand-held doppler ABI in patients with Type-2 diabetes.

**Methods:**

This prospective study was conducted at foot clinic of Baqai Institute of Diabetology and Endocrinology (BIDE), Baqai Medical University (BMU), a tertiary care unit, Karachi-Pakistan. The duration of study was February 2018 to March 2018. Patients with Type-2 diabetes attending the outpatient department (OPD) of foot clinic, irrespective of their symptoms were included. Baseline demographic, anthropometric measurements and biochemical parameters were recorded. The ABI was calculated with both devices by an automated oscillometric machine and standard hand-held doppler with the same investigator.

**Results:**

Total of 93 patients with Type-2 diabetes, 18 (19.4%) females and 75(80.6%) males were recruited. Mean age was 54.67±9.59 years and mean systolic/diastolic blood pressure was 131.38±20.2/ 80.36±10.23mmHg. Most of the patients had poor glycemic control at presentation with a mean HbA1c of 9.56±2.44%. Mean standard handheld doppler ABI and automated oscillometric ABI was 1.28±1.08 and 1.07±0.23 for right foot (mean difference = 0.21; P= 0.075), and 1.14±0.45 and 1.1±0.25 for left foot (mean difference =0.04; P=0.434), respectively. Similarly, sensitivity and specificity between two modalities was observed 60% and 93.90% for right foot, meanwhile, 60% and 97.40% for left foot, respectively.

**Conclusion:**

An automated oscillometric method is comparable with standard handheld-doppler method. It is cost effective, convenient and less time consuming, can be widely used to measure ABI without special training.

## INTRODUCTION

Peripheral arterial disease (PAD) is caused by partial or complete occlusion of limb arteries due to atherosclerosis. The concurrent occurrence of PAD was found to be markedly high in patients with Type-2 diabetes.^1^ Patients having PAD are more likely to suffer with foot ulcers and amputations, myocardial infarction, stroke or death as compared to patients who do not.^2^ Most of the individuals are asymptomatic and there is a slow and gradual progression of disease.^3^ Data on prevalence and outcome of Middle-Eastern patients with PAD is limited. In Pakistan, the prevalence of PAD in patients with Type-2 diabetes was 31.6%.^3^ While, the prevalence of PAD in American and Asian diabetic population was also estimated to be 9.5% and 3.2-11.7%, respectivey.^1^

For screening PAD, the ankle-brachial index (ABI) has been recognized as an accurate and reliable marker.^4^ Many studies have shown the value of ABI as a diagnostic tool for PAD and is considered as a simple and non-expensive method as compared to angiography.^5^ By convention, the ABI is the ratio of the highest systolic pressure at the ankle to the highest systolic pressure measured in the arms.^6^ A ratio of 0.9 or lower by doppler ultrasound confirms 50% or greater stenosis in one or more major vessels with 95% sensitivity and 100% specificity.^7^ However, the diagnostic accuracy of oscillometric ABI versus doppler ABI was shown controversial in Herráiz-Adillo A et al study.^8^ According to the clinical situation of patients with diabetes, ABI could be used but values should be interpreted with precision.^9^ However, this relatively simple calculation is rarely performed because of lack of equipment, the time required to do the procedure and technical difficulties involved in making measurements.

Recently, automatic devices have been developed to overcome and simplify ABI measurement procedures which appears to be convenient and as useful compared with hand held doppler.^10^,^11^ One of an automated method known as automated oscillometric technique used to measure ABI, and can be considered as a screening tool to identify high risk population.^12^ Due to its simplicity, easy to perform and not require special training, it can be suitable for screening of PAD in the community.^4^,^13^ Therefore, the main aim of our study was to compare the two modalities by measuring the difference between ABI using an automated oscillometric machine measurement as compared to standard hand-held doppler ABI on same patients with Type-2 diabetes, irrespective of their symptoms, either they have PAD or not.

## METHODS

This prospective study was conducted at Baqai Institute of Diabetology and Endocrinology (BIDE), Baqai Medical University (BMU), a tertiary care unit in Karachi- Pakistan. Patients with Type-2 diabetes, which itself is strong risk factor for PAD, attending outpatient department (OPD) of foot clinic, irrespective of their symptoms were included in our study. The duration of study was between February 2018 to March 2018. Ethical approval was obtained by Institutional Review Board (IRB) of BIDE. (BIDE/IRB/MR.ABZAFAR/06/11/18/0081). All patients provided written informed consent prior to participation in the study. Patients with any history of previous bypass surgery or angioplasty, any major amputations on the lower or upper limbs, marked edema of one or both feet, and atrial fibrillation were excluded from this study.

A proforma was prepared to maintain uniformity of the study. All measurements were obtained from the patients, avoiding smoking, heavy exercise and drinking alcohol or caffeinated beverages for at least two hours before the examination. Baseline demographic and anthropometric parameters including gender, age, duration of diabetes, family history of diabetes, body mass index (BMI), hypertension and smoking habit were noted. Biochemical parameters including glycated hemoglobin A1c, lipid profile and serum creatinine were also recorded. Detailed systemic examination along with palpation for diminution or absence of dorsalis pedis and posterior tibial pulses in both the limbs was performed. The ABI was calculated by recording systolic blood pressure in the supine position starting with the right arm, right leg, left leg, and left arm by using automated oscillometric and standard handheld doppler method for each patient. Blood pressure was repeated in the limbs, whenever there was an error recorded on the apparatus, with the same investigator.

ABI was calculated by dividing the highest value obtained at each ankle by the highest of the arm values. For definition of PAD, the lower value of both the left and right legs was considered. Normal cut-off values for ABI, adopted by most studies and by the accepted guidelines of cardiology societies are between 0.9 and 1.4. An abnormal ankle-brachial index below 0.9 is a powerful independent marker of cardiovascular risk.^14^ Glycemic control was assessed by HbA1c using HPLC method on BIO RAD D-10. To determine triglycerides, GOD- PAP method on Selectra Pro S a fully automated analyzer was used. Serum total cholesterol was analyzed by Chod-Pap method on Selectra Pro S (a fully automated analyzer). Homogeneous enzymatic colorimetric method was used for high density lipoprotein (HDL)–cholesterol and direct method used for low density lipoprotein (LDL)-cholesterol measurement.

Height was measured to the nearest 0.1cm with subject standing upright and weight was measured with a portable weighing scale to the nearest 0.1 kilogram (kg). Body mass index (BMI) was measured as the ratio of weight (kg) to height squared (m^2^). Blood pressures of the participants were monitored by mercury sphygmomanometer in a sitting position by using standard method. Hypertension was defined as blood pressure ≥130/85mmHg.^15^

### Statistical Analysis

All calculations were performed using statistical package for social science (SPSS) version 20. The data was expressed as mean ± SD and percentages. Paired t-test was applied to test the mean difference between standard hand held doppler ABI and automated oscillometric ABI. Statistical significance was defined as P < 0.05.

## RESULTS

Total of 93 patients with Type-2 diabetes, 18 (19.4%) females and 75(80.6%) males were included during the study period. Mean age of participants was 54.6±9.59 years and mean duration of diabetes was 13.9±8.5 years. Family history of diabetes was found in 45(75%) and smoking habit in 5(8.3%) patients. Mean BMI was 26.6±5.6 kg/m^2^ and mean systolic/diastolic blood pressure was 131.3±20.2/ 80.3±10.2mmHg. Most of the patients had poor glycemic control at presentation with a mean HbA1c of 9.5±2.4%. Mean values of total cholesterol, triglycerides, LDL (low-density lipoprotein) cholesterol, and HDL (high-density lipoprotein) cholesterol of study cases were 135.9±42.7mg/dl, 158±110.2 mg/dl, 76.5±37.3mg/dl, and 29.2±10.8mg/dl, respectively. Mean serum creatinine was 1.32±0.63 mg/dl (Table-I).

**Table I:** Baseline and biochemical characteristics of patients with Type-2 diabetes.

Parameters		Mean±SD or n (%)
n		93
Age (years)		54.67±9.59
Gender	Female	18(19.4%)
	Male	75(80.6%)
Body mass index (BMI, kg/m^2^)		26.69±5.6
Marital status	Married	56(94.9%)
	Single	3(5.1%)
Smoking habit	Yes	5(8.3%)
Systolic blood pressure (mmHg)		131.38±20.2
Diastolic blood pressure (mmHg)		80.36±10.23
Family history of diabetes	No	15(25%)
	Yes	45(75%)
Duration of diabetes (years)		13.97±8.5
HbA1c (%)		9.56±2.44
Total cholesterol (mg/dl)		135.97±42.72
High-density lipoproteins (mg/dl)		29.21±10.84
Low-density lipoprotein (mg/dl)		76.52±37.31
Triglycerides (mg/dl)		158±110.26
Creatinine (mg/dl)		1.32±0.63

Data presented as mean ± SD or n (%)

### Comparison of an automated oscillometric ABI and standard hand-held Doppler ABI

The mean values of ABI measured by standard hand held doppler for left and right foot was 1.14±0.45 and 1.28±1.08, while an automated oscillometer ABI was 1.1±0.25 and 1.07±0.23, respectively. The difference between an automated oscillometric ABI and standard handheld doppler ABI was not significant for right foot (mean difference = 0.21; P= 0.075) as well as for left foot (mean difference = 0.04; P=0.434) (Table-II).

**Table II:** Comparison of an automated oscillometric ABI and standard hand-held doppler ABI.

Parameters			Mean±SD	Mean difference	P-value
Left foot	Standard hand-held doppler ABI	1.14±0.45	0.04	0.434
	Automated oscillometric ABI	1.1±0.25		
Right foot	Standard hand-held doppler ABI	1.28±1.08	0.21	0.075
	Automated oscillometric ABI	1.07±0.23		

Data presented as Mean ± SD,

P-value<0.05 considered to be statistically significant, Paired T test was applied.

**Fig. 1:**
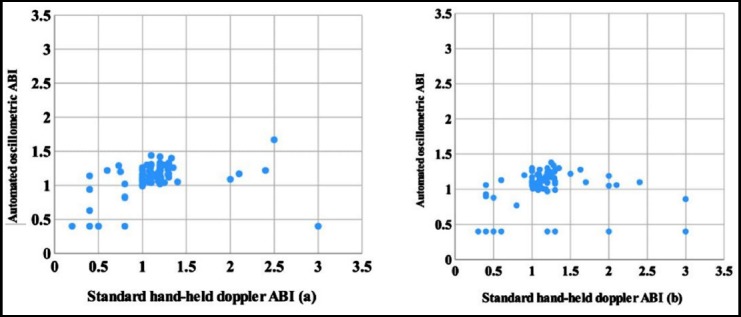
Scattered plot between an automated oscillometric ABI standard hand-held Doppler ABI for left foot (a) and right foot (b).

The scattered plot of an automated oscillometric ABI and standard hand-held doppler ABI for left and right foot was shown in Fig. 1(a) and 1(b), respectively.

The sensitivity and specificity between two modalities was found 60% and 93.90% for right foot, meanwhile, 60% and 97.40% for left foot, respectively. For left foot, positive predictive value (PPV) was observed 81.81% and negative predictive value (NPV) was 92.68%, similarly, for right foot was 54.50% and 95.12%, respectively (Table-III).

**Table III:** Sensitivity, specificity, positive predictive value and negative predictive value between two modalities (oscillometric ABI and hand-held doppler ABI)

Automated oscillometric ABI	Standard hand-held doppler ABI					

	Left Foot			Right Foot		

	<0.9	≥0.9	Total	<0.9	≥0.9	Total
<0.9	9	2	11	6	5	11
≥0.9	6	76	82	4	78	82
*Total*	15	78	93	10	83	93
Sensitivity	60%			60%		
Specificity	97.40%			93.90%		
Positive predictive value (PPV)	81.81%			54.50%		
Negative predictive value (NPV)	92.68%			95.12%		

## DISCUSSION

Our study showed that an automated oscillometric method is comparable with standard handheld-doppler method. The main finding of our study is the insignificant difference between an automated oscillometric method and standard hand held doppler method for both right and left foot, respectively. These results are similar to MacDougall AM et al study, that found an automated oscillometric ABI is feasible for measuring ABI.^16^

In Takahashi I et al. study, the validity and reliability of automated oscillometric ABI values for diagnosing PAD is controversial.^17^ Although the gold standard is doppler-derived ABI, but, the automated oscillometric-ABI measurement has its own advantages like being simple and convenient with low cost for PAD screening. However, Ma J et al study from China validated the concordance between oscillometric ABI and doppler ABI.^11^ Similarly, using automated oscillometric method was highly consistent with that by standard handheld doppler method, to detect a larger number of PAD cases reported by Ma J et al study.^11^ Using an automated oscillometric method is simple, cost effective, less time consuming and can be widely used for measuring ABI without special training. However, standard handheld doppler ABI measurement is time-consuming and require specific skills to measure ABI.^8^ On the other hand, oscillometric measurement of ABI had good correlation with doppler ultrasound measurements with little difference between the two different machines tested. Patients suspected of high risk for atherosclerosis, have negative examinations require further testing.^18^

In this study, almost all patients were on statin as they were diagnosed cases of Type-2 diabetes. Less than 20 patients were on amlodipine with angiotensin converting enzyme inhibitors (ACE inhibitors) or angiotensin II receptor blockers (ARBs) for hypertension. This data had no impact on our study of comparing two modalities irrespective of whether they had PAD or not, as their comparison was statically insignificant. The standard hand held doppler method is operator dependent and the intrinsic bias do exist during measurements, while as the automated oscillometric-ABI value is not influenced by the operator and the results usually are more reliable. Similarly, the time needed for standard hand held doppler ABI was longer than that for oscillometric-ABI methods due to the necessity of additional steps with doppler, such as pulse palpation, the application of gel, signal viewing, and operational levels. Abraham P et al reported that to identify the optimal method of ABI, calculation for predicting cardiovascular events and mobility loss is encouraged.^19^ To date, insufficient evidences are available to support substituting doppler machines with oscillometric devices for determination of ABI reported by e Silva G et al study.^20^ More studies are needed to be done to explore potentially easier and faster alternative methods for ABI measurement that would likely be implemented more broadly in primary care. Standards of accreditation are necessary for the ABI measurement devices, using methods other than doppler devices (e.g, oscillometric methods).

A major oscillometer drawback is that pressure difference between both methods in ankle varies significantly according to the pressure range, with a potential loss of sensitivity. Herráiz-Adillo Á et al studied that by using oscillometer technique, this under or overestimation in ABI especially happens at extreme values of ABI, and does not affect the agreement on the area of discrimination (0.9).^8^ Therefore, our data support that oscillometric device seems to be a valid technique to diagnose PAD, but not its severe degree. Another drawback of oscillometric method is its lack of ability to measure low pressures in the ankle in comparison to doppler, which despite giving useful information either low ABI or calcification in these patients. Therefore, to monitor patients with severe PAD, it would invalidate the use of this device. Despite these drawbacks, oscillometric ABI might be a useful tool to screen and diagnose PAD.

Ma J et al study based on nondiabetic subjects, suggest that an automated oscillometric ABI measurement is a reliable and practical alternative to the conventional doppler measurement for the detection of PAD,^11^ but our objective of the study was to involve diabetic patients to validate automated oscillometric ABI method. Further studies are needed to be performed, as this study was a single-center study and the sample size was not enough. The apparatus is not standard one. But, due to its simplicity this method scores over the standard method.

### Limitations of the study

Firstly, in this study we used doppler ABI as reference standard due to the fact that it is considered as the non-invasive gold-standard technique, but there are always variations in its readings as it is operator dependent and needs expert to perform it which are not available easily. Secondly, due to the presence of a unique examiner, it was not possible to blind the measurements between oscillometric and doppler technique. As the oscillometric technique is fully automatic technique, a bias is only permissible-diagnostic review bias-when the oscillometric technique was performed prior to doppler.

## CONCLUSION

This study concludes that an automated oscillometric method is comparable with standard handheld-doppler method. It is cost effective, convenient and less time consuming, can be widely used to measure ABI without special training.

### Author’s contribution

**SZK:** Concept, design, literature search, interpretation of data, prepared the manuscript.

**ABZ:** Concept, design, interpretation of data, edited and approved the manuscript.

**NW:** Literature search, edited and approved the manuscript.

**ZM:** Concept, design, edited and approved the manuscript.

**MSH:** Edited and approved the manuscript.

**AF:** Interpretation of data, edited and approved the manuscript.
